# *SNORD99* promotes endometrial cancer development by inhibiting GSDMD‐mediated pyroptosis through 2'‐O‐methylation modification

**DOI:** 10.1111/jcmm.18500

**Published:** 2024-06-22

**Authors:** Jing‐yuan Xian, Wu Wu, Xi Chen, Hai‐juan Bao, Song Zhang, Xiu‐Jie Sheng, Shuo Chen

**Affiliations:** ^1^ Department of Obstetrics and Gynecology, Guangzhou Key Laboratory of Targeted Therapy for Gynecologic Oncology, Guangdong Provincial Key Laboratory of Major Obstetric Diseases, Guangdong Provincial Clinical Research Center for Obstetrics and Gynecology, Guangdong‐Hong Kong‐Macao Greater Bay Area Higher Education Joint Laboratory of Maternal‐Fetal Medicine The Third Affiliated Hospital of Guangzhou Medical University Guangzhou China; ^2^ Department of Gynecologic Oncology Research Office The Third Affiliated Hospital of Guangzhou Medical University Guangzhou China; ^3^ Department of Thoracic Surgery The First Hospital of China Medical University Shenyang China

**Keywords:** 2'‐O‐methylation, endometrial cancer, GSDMD, pyroptosis, small nucleolar RNA

## Abstract

Eukaryotic cells possess multiple mechanisms of self‐destruction, including pyroptosis and necroptosis. Pyroptosis is a type of programmed cell death characterized by cellular rupture and linked to inflammation. SnoRNA, a small non‐coding RNA in the nucleolus, can dysregulate specific RNAs through 2'‐O‐methylation, contributing to tumorigenesis. Our StarBase and qRT‐PCR analysis revealed *SNORD99* upregulation in endometrial cancer (EC) tissue compared to normal tissue, suggesting its role in pathogenesis. *SNORD99* overexpression enhanced migration and proliferation of EC cells, while ASO‐mediated suppression reduced malignant cell spread and division. RNA‐seq and base‐comparing analysis identified GSDMD's differential expression upon *SNORD99* overexpression, forming the *SNORD99*‐FBL RNP complex. RTL‐P experiments showed *SNORD99* increased GSDMD's 2'‐O‐methylation. *SNORD99* reduced GSDMD, caspase‐1, and NLRP3 protein levels, implicating its role in pyroptosis. Optical and electron microscopy confirmed enhanced pyroptosis features. In summary, *SNORD99* modifies GSDMD via 2'‐O‐methylation, suppressing pyroptosis and promoting EC progression. Developing pyroptosis‐inducing drugs may offer new cancer treatment avenues.

## 1 INTRODUCTION

Endometrial cancer (EC) is a malignancy arising from the epithelial cells lining the uterus with increasing morbidity and mortality worldwide.^1^ In 2020, EC sickened approximately 417,336 patients and became the most frequently diagnosed malignancies in women.^2^ Although more than 60% of patients suffer early clinical symptoms and the survival rate over a five‐year period of early‐stage cases is about 81%, the five‐year survival rates of advanced stage cases such as IVA and IVB EC stages are only 17% and 15%, respectively.^3^ The occurrence of EC may be related to the mutations of multiple proto‐oncogenes or tumour suppressor genes. Thus, the molecular mechanisms that drive the onset and development of EC is crucial for advancing the discovery of efficient approaches for timely detection, diagnosis and management.

SnoRNA, a non‐coding RNA molecule, is present in eukaryotic cells and is found throughout the nucleolus. The length of these molecules ranges from 60 to 300 nucleotides. It can form complexes with ribonucleolar nucleoproteins to create snoRNPs. These snoRNPs are involved in various cellular processes, including the processing of ribosomal RNA, regulating RNA splicing and translation and responding to oxidative stress.^4^ The snoRNA possesses conserved structural components, which can be categorized into three groups: box C/D snoRNA (SNORD), box H/ACA snoRNA (SNORA) and MRP RNA.^5^ Emerging evidence has revealed that SNORDs have been shown to modulate gene expression by complementary base pairing and forming snoRNP complexes with ribonucleolar proteins (RNPs), leading to 2'‐O‐methylation of rRNA, tRNA, mRNA, snRNA and other RNAs.^6^, ^7^ In 2022, Wen et al. established that *SNORD15B* promoted the development of EC by regulating the TRIM25/P53 complex.^8^ Chen et al. found that SNORA70E promotes the onset and progression of ovarian cancer by pseudouridylation modification of RAP1B and PARPBP.^9^ In addition, Lu et al. discovered that *SNORD104* promoted EC through the regulation of PARP1 2'‐O‐methylation.^10^ Furthermore, Bao et al. evidenced that the modulation of 2'‐O‐methylation modifications by *SNORD89* is implicated in the pathogenesis of EC, specifically in the regulation of Bim.^11^ These results suggested that SNORDs participate in the tumorigenesis and progression of endometrial carcinoma.

Pyroptosis is a type of regulated cell death that is characterized by inflammation and is induced by a group of caspases known as pyroptosis‐associated caspases. It can be triggered by pathological factors, such as infections, cell damage and malignant tumours, and is characterized by swelling and rupture of the cell membranes and the release of cellular contents. Gasdermin family proteins are key molecules participating in pyroptosis.^12^, ^13^, ^14^ Gasdermin D (GSDMD), a 53‐kDa cytoplasmic protein, is a key substrate for inflammatory caspases (such as caspase‐1, ‐4, ‐5, and ‐11) to trigger pyroptosis.^15^, ^16^ When pathogens enter cells, lipopolysaccharide (LPS) is released to activate caspase‐11, ‐4, and ‐5 or caspase‐1. Upon activation, inflammatory caspases cleave GSDMD and caspase‐1 can also increase the secretion of mature IL‐1β. The structural composition of GSDMD includes a pore‐forming domain situated at the N‐terminus, a regulatory domain at the C‐terminus, and a linking region positioned at the center.^17^, ^18^ Following cleavage, the N‐terminal portion of GSDMD is liberated and undergoes activation. Once activated, GSDMD‐N binds with lipids present in the plasma membrane, ultimately results in the development of large pores. Cell death can occur when extracellular fluid and calcium enter cells through membrane pores, activating the release of intracellular contents, such as cytokines and danger‐associated molecular patterns (DAMPs), as well as inflammatory substances.^19^

Our analysis of the StarBase (https://rnasysu.com/encori/), an open platform for analysing data include lncRNAs, miRNAs, snoRNAs, mRNAs, etc. related to 32 cancer types integrated from the TCGA project, revealed that the *SNORD99* expression showed a significant increase in EC tissue compared to normal endometrial tissue. *SNORD99* is a member of the box C/D snoRNAs found within the snoRNA, specifically situated at 1p35.3 and known as HBII‐420 as well. Our preliminary experiments also found that the expression levels of GSDMD showed a significant decrease after *SNORD99* overexpression by Ishikawa cells, suggesting that *SNORD99* may regulate the pyroptosis signalling pathway, thereby promoting EC occurrence and development. However, no research has been conducted on the regulation of the mechanism of pyroptosis by snoRNA. Thus, we conducted experiments both in laboratory settings and within living organisms for the first time to investigate the potential molecular mechanism of *SNORD99* involved in pyroptosis‐related EC tumorigenesis and progression.

## 2 MATERIALS AND METHODS

### 2.1 Bioinformatics analysis

The expression levels of *SNORD99* in specimens of normal endometrial and EC tissues were obtained from StarBase (https://rnasysu.com/encori/).

### 2.2 Human tissue specimens

A total of 30 samples of normal endometrial tissue and 126 samples of EC tissue were obtained from individuals who received surgical intervention at the Third Affiliated Hospital of Guangzhou Medical University. Before the surgery, all the individuals involved in the study did not receive any radiotherapy or chemotherapy treatments. The specimens were rapidly cryopreserved in liquid nitrogen and maintained at −80°C until further analysis. The study protocol was approved by the hospital's ethics committee (no. 2020066), and all patients were informed and provided written consent.

### 2.3 Cell culture and transfection

Ishikawa, HEC1A, HEC1B and HESC (human endometrial stromal cells) were obtained from ATCC (Manassas, VA, USA) and Jennio Biotech (Guangzhou, China). The DMEM (HyClone, Logan, UT, USA) culture medium was used for HEC1B cells, while RPMI‐1640 medium (HyClone) was utilized for the cultivation of HEC1A and Ishikawa cells. The culture medium was prepared by adding 10% FBS (Gibco, Grand Island, NY, USA) and 100 U/mL of penicillin–streptomycin (Gibco) to the media. The incubation temperature was 37°C and with 5% CO_2_. Transfection of plasmids and Antisense oligonucleotide (ASOs) was carried out using Lipofectamine 3000 (Invitrogen, Carlsbad, CA, USA). The *SNORD99*‐overexpressing plasmid sequence was as follows: (ACTGGTCCAGGATGAAACCT AATTTGAGTGGACATCCATG GATGAGAAATGCGGATATGG GACTGAGACCAGCTCCTAGG). The target gene sequence for ASO (Ruibo, Jiangsu, China) was ATGCGGATATGGGACTGAGA.

### 2.4 Cell viability experiment

After cell adherence, cells were plated in 96‐well plates at a concentration of 2000 cells per well and transfected with the appropriate agents. At designated time points (0, 24, 48 and 72 h), each well was treated with 10 μL of Cell Counting Kit‐8 (CCK‐8, Yeasen, Shanghai, China) and incubated for an additional 2 h at 37°C. The optical density (OD) of each well was detected at 450 nm using a microplate reader. The experimental procedure was carried out in triplicate.

### 2.5 RNA stability

Cells overexpressing or knocking down *SNORD99* were seeded into 6‐well plates. When the cell growth rate reached 80%, 5 μg/mL actinomycin D (Act D, Sigma, St. Louis, MO, USA) was added to each well, and cells were collected at 0, 3 and 6 h, then lysed using TRIzol reagent and total RNA was extracted, and finally GSDMD mRNA levels were detected by qPCR.

### 2.6 Wound healing

Inoculate 600,000 cells into a 6‐well culture plate. The next day, using the tip of a 200 μL pipette, create two perpendicular scratches in each well. Subsequently, wash the cells in the wells three times with PBS and 2 mL of serum‐free medium was added to each well. Record the cell migration at 0 and 48 h, and analyse the wound area using ImageJ software (National Institutes of Health, Bethesda, Maryland) to determine the rate of cell migration.

### 2.7 Cell migration assay by transwell

A total of 50,000 cells overexpressing or knocking down *SNORD99* were seeded in the upper chamber of the Transwell chamber with 200 μL serum‐free medium, then 600 μL of serum‐containing medium was added to the lower chamber. After 48 h of incubation in the incubator, the chambers were removed and washed with PBS, then the cells were fixed with paraformaldehyde for 20 min, and finally the cells were stained with crystal violet for 20 min. Cells were photographed under a microscope (Olympus, Tokyo, Japan) and counted using ImageJ.

### 2.8 Real‐time quantitative reverse‐transcription PCR

RNA was isolated from the cells using an RNA rapid purification kit, and random primers from the Fast Reverse Transcription Kit (Tiangen, Beijing, China) were used to generate complementary cDNA. The synthesized cDNA was utilized as a template for quantitative real‐time PCR (qRT‐PCR) with the Hieff® qPCR SYBR Green Master Mix (Yeasen, Shanghai, China). The 2^−ΔCt^ method was employed to determine the expression level of the target gene by contrasting the Ct value of the target gene with that of a reference gene (U6).

### 2.9 RNA immunoprecipitation analysis

Cell scraping was employed to harvest the cells. The cells were resuspended in an equal volume of RNA immunoprecipitation (RIP) lysis buffer. After centrifugation, the supernatant was collected and incubated with magnetic beads conjugated with anti‐human FBL antibody and normal rabbit IgG (negative control) in RIP buffer, then washed twice with a magnetic rack, and the supernatant was discarded. Then, total RNA was extracted from the pellet by adding 1 mL of TRIzol reagent (Takamura, Shibuya, Japan). Chloroform (200 μL) was added to the mixture, mixed and centrifuged at 12,000 rpm for 20 min. The supernatant was removed and an equal volume of isopropanol was added to precipitate the RNA. After centrifuging again at 12,000 rpm for 20 min, the supernatant was discarded and the precipitate was washed with 75% ethanol. After the alcohol has completely evaporated, the precipitate was dissolved with DEPC water. Spectrophotometry (Thermo Fisher Scientific, Shanghai, China) was used to determine the concentration of total RNA. Then, total RNA was converted into cDNA by employing reverse transcriptase alongside random primers following the protocol provided by manufacturer (Yeasen). This cDNA served as the template for qRT‐PCR, employing the SYBR Prime X Ex‐TAQ Kit (Takara Bio Inc., Shiga, Japan). Finally, according to the $2^{-\text{ΔCt}}$ method, the Ct value of the target gene and the reference gene (U6) was compared to calculate the relative expression of the gene.

### 2.10 Western blotting

The GSDMD antibody (20770‐1‐AP, diluted at 1:5000; Proteintech), NLRP3 (D4D8T, diluted at 1:800; Cell signal), caspase‐1 (D7F10, diluted at 1:800; Cell signal), and GAPDH (10494‐1‐AP, diluted at 1:10,000; Proteintech) were used to evaluate protein expression. To extract proteins from EC cells, PBS was used to wash the cells followed by lysis in RIPA lysis buffer supplemented with protease inhibitors. The lysates were then subjected to centrifugation at 12,000 rpm and 4°C for 20 min, and the protein concentration was measured with a BCA Protein assay Kit (P0009; Beyotime, Shanghai, China). Subsequently, protein samples were separated by electrophoresis on a 10% SDS‐polyacrylamide gel and subsequently transferred onto western blotting membranes (Amersham Hybond, Munich, Germany). The membranes were incubated in a blocking solution of 3% BSA in TBST at room temperature for 2 h, and primary antibodies specific to GSDMD, NLRP3 and caspase‐1 were added and incubated overnight at 4°C. A secondary antibody (1:10000, Proteintech) was added to the membrane on the second day and incubated at room temperature for 2 h, and GAPDH was used as the internal reference. The membranes were subjected to three washes with TBST after incubation and protein development was detected using a Ultra High Sensitivity ECL Substrate Kit (NcmECL UItra; Sliding Biotech, Shanghai, China). The protein bands were visualized using an enhanced chemical lighting system.

### 2.11 In vivo tumour formation experiment in nude mice

A subcutaneous tumour transmission detection kit was utilized in this experiment to induce tumour formation, female BALB/C nude mice were used in this study (*n* = 6/each group). The mice were maintained under conditions that were free of specific pathogens and were randomly assigned to different groups. Female mice that were 5 weeks old were subjected to subcutaneous injection of Ishikawa cells transfected with Plasmids carrying *SNORD99* or no‐load control suspended in 150 μL of FBS‐free medium to induce tumour formation. Weekly measurements were taken using vernier callipers to monitor the growth of the tumours and determine their volume. After 4 weeks, humane euthanasia was performed on the mice, and the tumours were excised and measured for size. This study was conducted with the approval of the relevant ethical committee.

### 2.12 Reverse transcription at low dNTP concentrations followed by polymerase chain reaction

To detect Nm of *SNORD99*, RT‐PCR was conducted using a 20‐μL reaction system comprising 5 μg of total RNA, different concentrations of dNTPs (TaKaRa), and 1 μL of specific reverse transcription primers for the target gene (PF) at a temperature of 65°C for a duration of 5 min. Subsequently, after the addition of 4 μL of 5 × M‐MLV Reverse Transcriptase buffer (PROMEGA), the mixture was subjected to incubation at 50°C for a duration of 1 h, 1 μL of 0.1 M DL‐Dithiothreitol (PROMEGA), and 1 μL of 40 u/μL RNase inhibitor (PROMEGA), followed by the incubation that was carried out at 70 C for a duration of 15 min. The SYBR Prime X Ex‐TAQ Kit (Takara) was employed for qRT‐PCR, using the cDNA as template. Ultimately, the gene's expression level was assessed through the 2^−ΔCt^ method by comparing the Ct value of the target gene in the experimental group with that in the control group at low concentrations.

### 2.13 EdU assay

Cells from the Ishikawa or HEC1B cell lines were cultured in 96‐well plates and treated with EdU assay kit (Thermo Fisher, USA) as per the manufacturer's instructions. Subsequently, the cells were stained and observed using a microscope (Olympus, Tokyo, Japan).

### 2.14 Immunofluorescence

Ishikawa/HEC‐1B cells were fixed onto 10‐mm circular glass slides with 4% paraformaldehyde for 15 min. Afterward, the cells were washed thrice using PBS and then exposed to 0.2% Triton X‐100 (w/v) in PBS for a duration of 15 min. Following three washes with PBS, the cells were subjected to a blocking procedure for 2 h using 5% bovine serum albumin (BSA). The cells were subjected to incubation with the primary antibody targeting GSDMD (20770‐1‐AP, 1:400, Proteintech, China) for an overnight duration at 4°C. Next, the cells were treated with the secondary antibody, Alexa Fluor 596‐labelled goat anti‐rabbit IgG (1:500, Beyotime, China), and incubated for 2 h. The fluorescence images were acquired using a Leica microscope.

### 2.15 Lactate dehydrogenase analysis

The release of lactate dehydrogenase (LDH) from cells into the culture supernatant is a commonly used indicator of cell damage and death. To measure LDH levels in the culture supernatants of EC cells that were exposed to LPS for 24 h, the non‐radioactive CytoTox 96 Cytotoxicity Assay Kit (Promega) was utilized as per the manufacturer's guidelines. All infection assays were carried out in serum‐ and antibiotic‐free media, and the results were analysed using a Synergy Mx plate reader (BioTek, USA).

### 2.16 Scanning electron microscopy (SEM)

HEC‐1B cells were grown on 10‐mm glass coverslips, rinsed twice with PBS, and fixed in 2.5% Gluta‐specific fixative for electron microscopy (Solarbio) for 1–2 h. The fixed cells were washed with PBS buffer, post‐fixed in 1% OsO_4_ for 30 min, and gradually dehydrated using an ethanol series. Next, the cells were critically dried, mounted on stubs, coated with a thin layer of conductive metal (gold and palladium) and examined under an SEM microscope (Hitachi‐S‐3400).

### 2.17 Data analysis

Data analysis and graphing were performed using GraphPad Prism v 8.01(244) (GraphPad Software, CA, USA). The data were presented as mean ± standard error of the mean, and statistical analysis was conducted using a two‐sample *t*‐test. A *p*‐value less than 0.05 was considered statistically significant.

## 3 RESULTS

### 3.1 The expression of *SNORD99* is upregulated in endometrial cancer tissues

**FIGURE 1:**
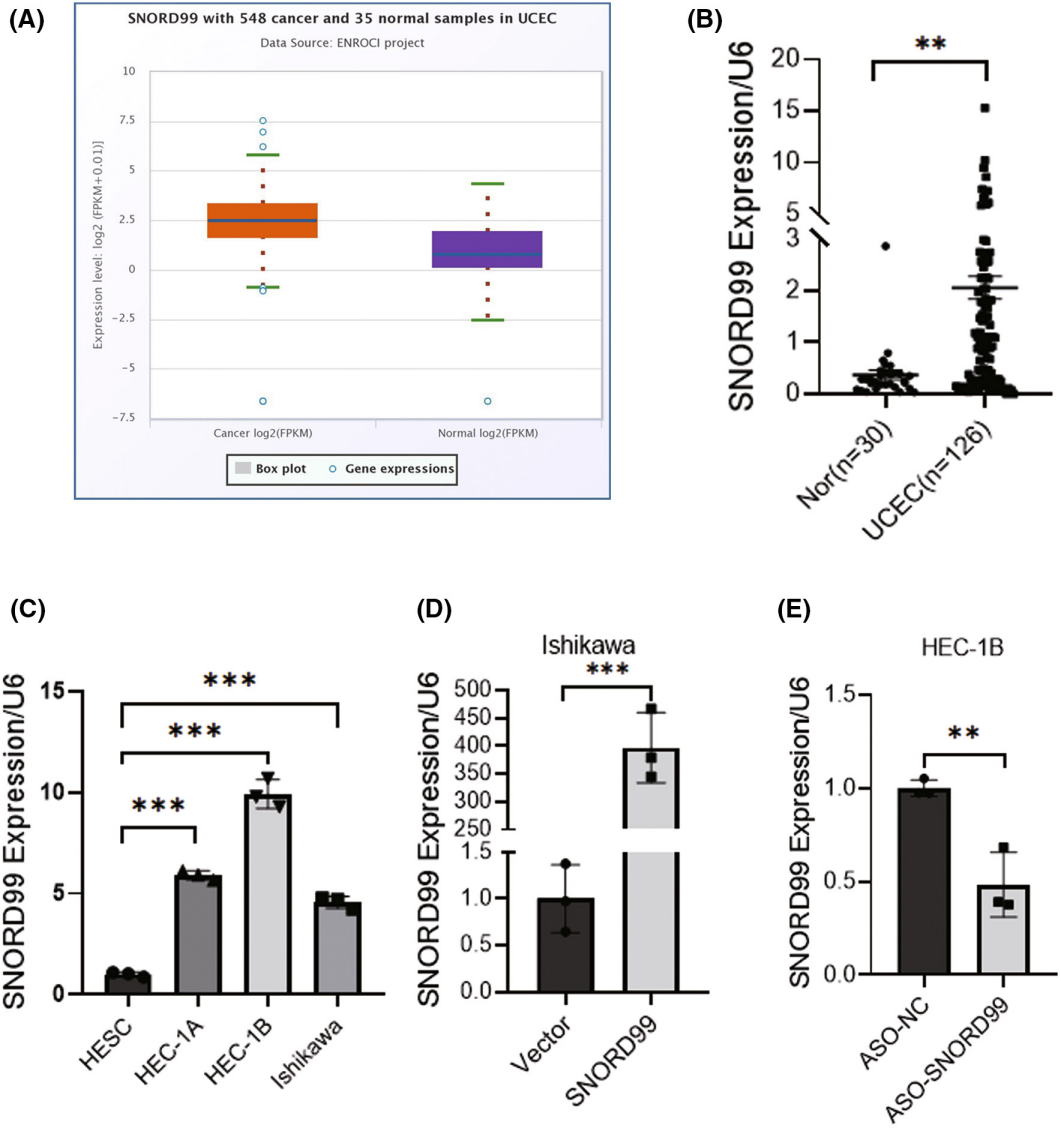
Gene expression of *SNORD99* in endometrial cancer (EC) tissues and cell lines. (A) Data from the StarBase databases. (B) *SNORD99* expression in EC tissues. (C) *SNORD99* expression in EC cell lines and human endometrial stromal cells (*n* = 3). (D) Transfection efficiency after overexpression of *SNORD99* (*n* = 3); (E) Transfection efficiency after downregulation of *SNORD99* by ASO (*n* = 3). Values are presented as mean ± SD. *p ≤ 0.05, **p ≤ 0.01 and ***p ≤ 0.001.

We analysed the StarBase, and found that the expression of *SNORD99* was markedly elevated in EC tissue in comparison with that in normal endometrial tissue (Figure 1A, *p < 0.05). We collected 30 normal endometrial tissue specimens and 126 EC tissue specimens, and performed qRT‐PCR on these samples. The results showed that the expression of *SNORD99* in EC tissues was significantly higher than that in normal endometrial tissues (Figure 1B, *p < 0.05). Additionally, qRT‐PCR results showed that *SNORD99* expression was highest in HEC‐1B cells and lowest in Ishikawa cells (Figure 1C, *p < 0.05). To further investigate, we overexpressed *SNORD99* in Ishikawa cells and used *SNORD99* ASO to knock down its expression in HEC‐1B cells. The transfection efficiency was confirmed by qRT‐PCR (Figure 1D,E, *p < 0.05).

### 3.2 Overexpression of *SNORD99* promotes cell proliferation and migration of EC cells

**FIGURE 2:**
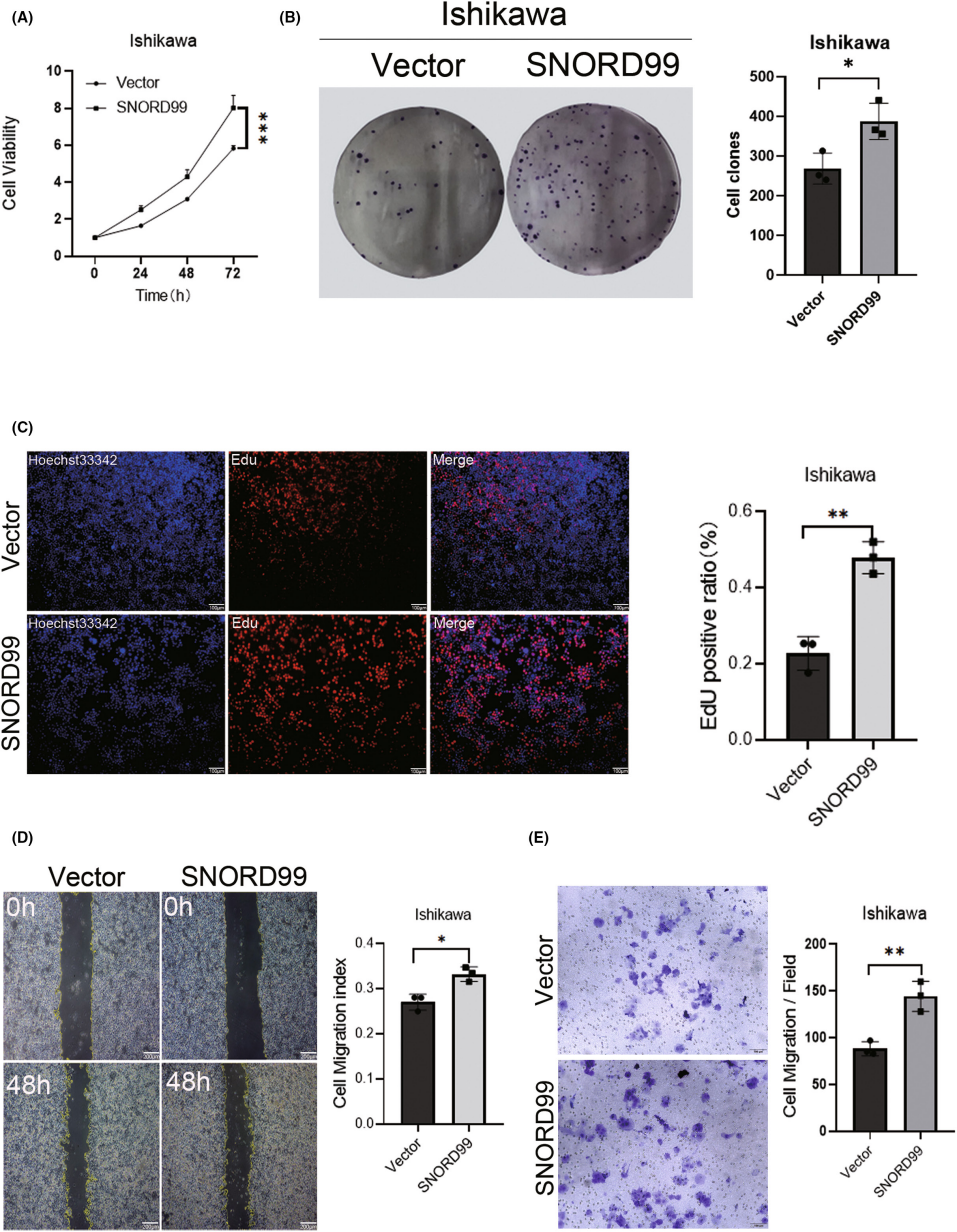
Cellular function of *SNORD99* overexpression in vitro. *SNORD99* overexpression (A) induced cell viability (*n* = 3), (B) promoted cell colony formation (*n* = 3), (C) induced EdU fluorescence labelling for cell proliferation and (D & E) induced cell migration (*n* = 3). Values are presented as mean ± SD. *p ≤ 0.05, **p ≤ 0.01 and ***p ≤ 0.001.

After *SNORD99* overexpression in Ishikawa cells, we performed cell counting, plate clone formation, wound‐healing and cell proliferation EdU‐fluorescent labelling experiments to determine the function of *SNORD99* in the proliferation and migration of EC cells. The results showed that *SNORD99* overexpression promoted cell proliferation (Figure 2A–C, *p < 0.05) and enhanced cell migration ability (Figure 2D,E, *p < 0.05).

### 3.3 Knockdown of *SNORD99* inhibits the malignant tumour characteristics of EC cells

**FIGURE 3:**
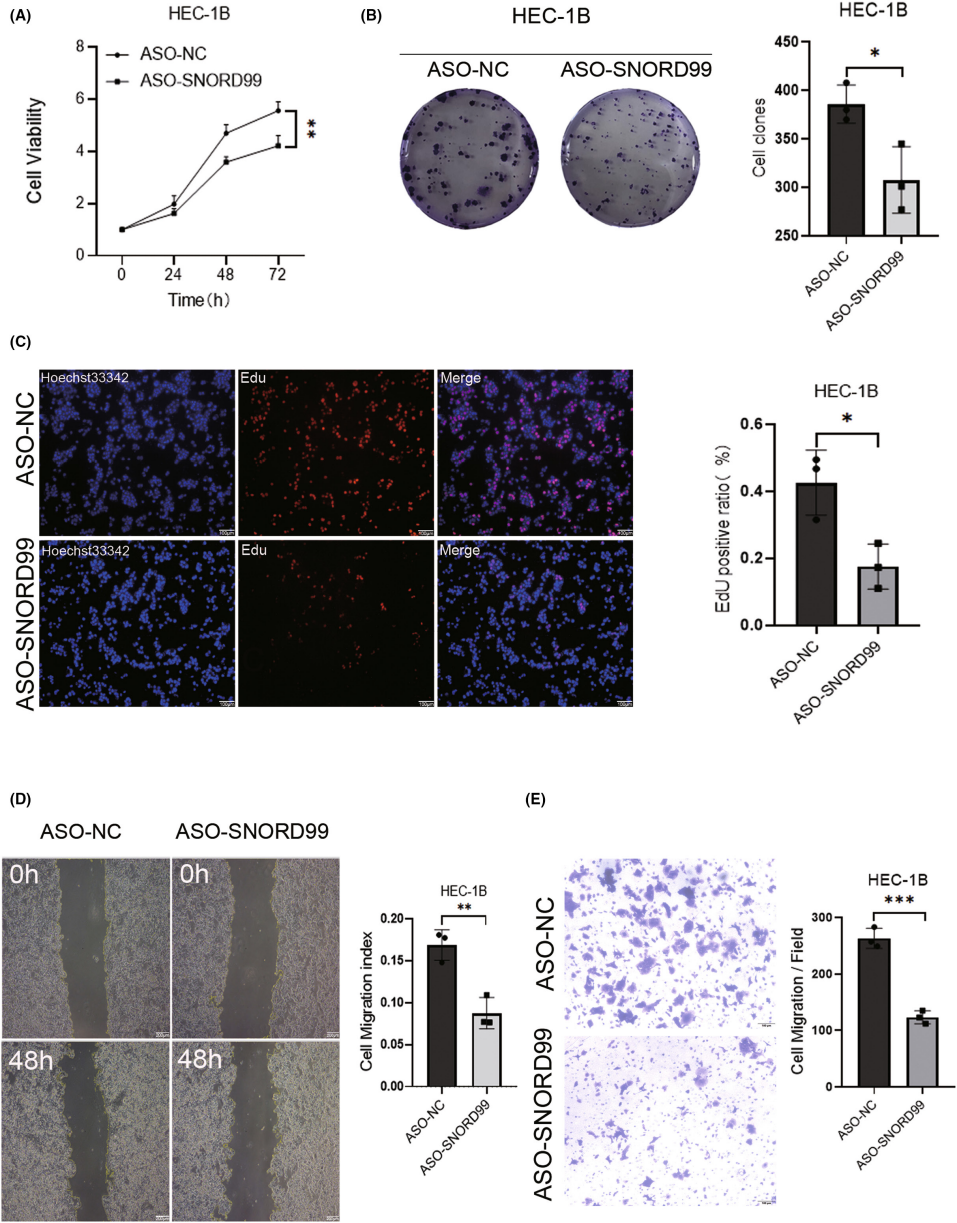
Cellular function of knockdown *SNORD99* in vitro. Downregulation of *SNORD99* (A) reduced cell viability (*n* = 3), (B) inhibited cell colony formation (*n* = 3), (C) inhibited EdU fluorescence labelling for cell proliferation and (D & E) reduced cell migration (*n* = 3). Values are presented as mean ± SD. *p ≤ 0.05, **p ≤ 0.01 and ***p ≤ 0.001.

After knocking down *SNORD99* by transfection with ASO‐*SNORD99* in HEC‐1B cells, we performed cell function experiments. *SNORD99* knockdown inhibited cell proliferation (Figure 3A–C, *p < 0.05) and migration (Figure 3D,E, *p < 0.05) in comparison to the negative control group.

### 3.4 In vivo nude mouse tumorigenicity assay of *SNORD99*

**FIGURE 4:**
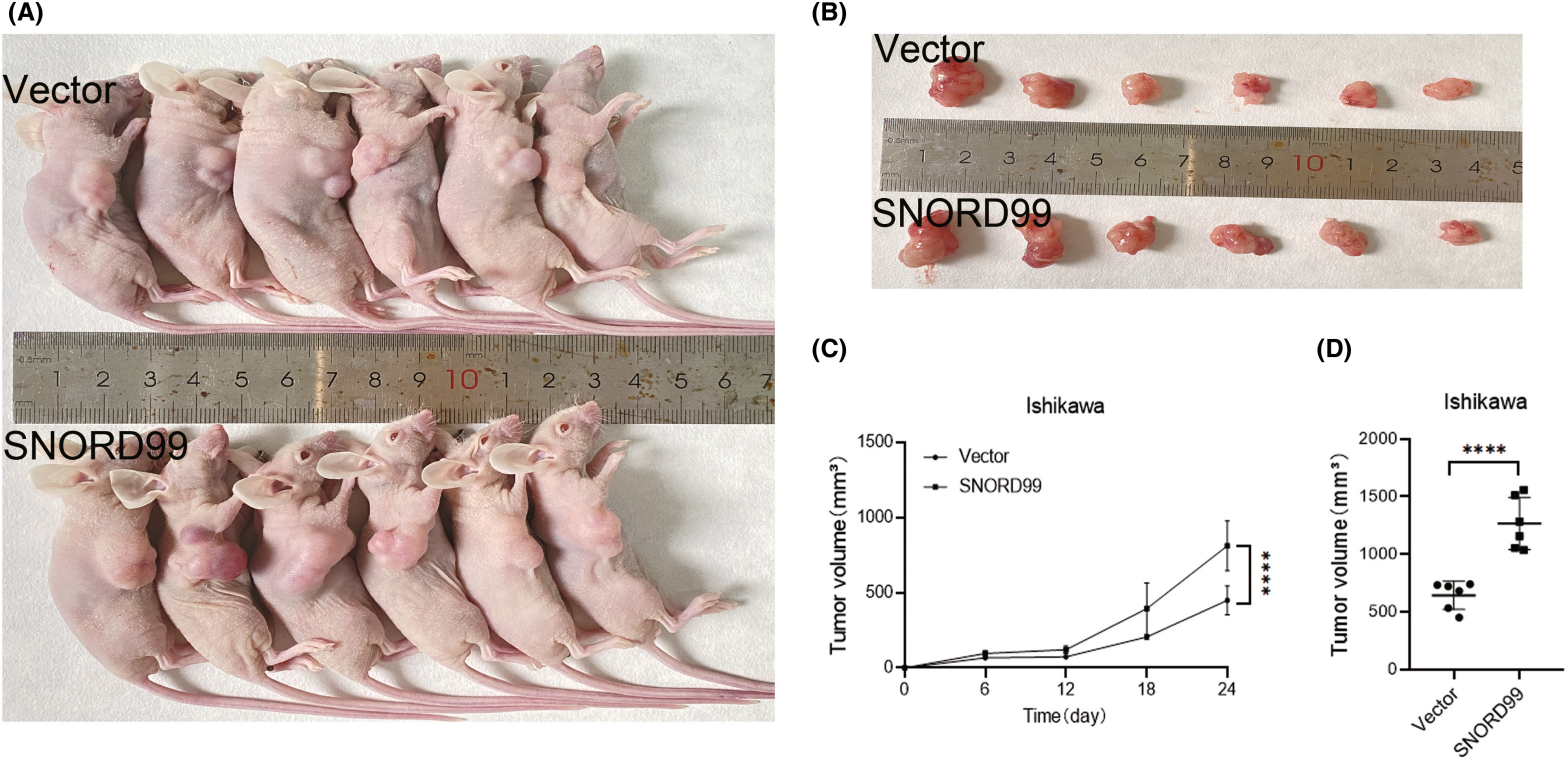
Tumour formation effect of overexpressed *SNORD99* in vivo. (A–C) Mice injected with *SNORD99*‐overexpressing cells were more tumorigenic than controls. (D) The tumour volume was larger (*n* = 6). Values are presented as mean ± SD. ****p ≤ 0.0001.

To investigate the in vivo effects of *SNORD99*, tumour formation experiments were conducted in nude mice (Figure 4A). The findings indicated that Ishikawa cells overexpressing *SNORD99* lead to faster tumour formation (Figure 4C, *p < 0.05) and larger tumour volumes compared to the control group (Figure 4B,D, *p < 0.05).

### 3.5 *SNORD99* inhibits the expression of pyroptosis‐related proteins in EC cells

**FIGURE 5:**
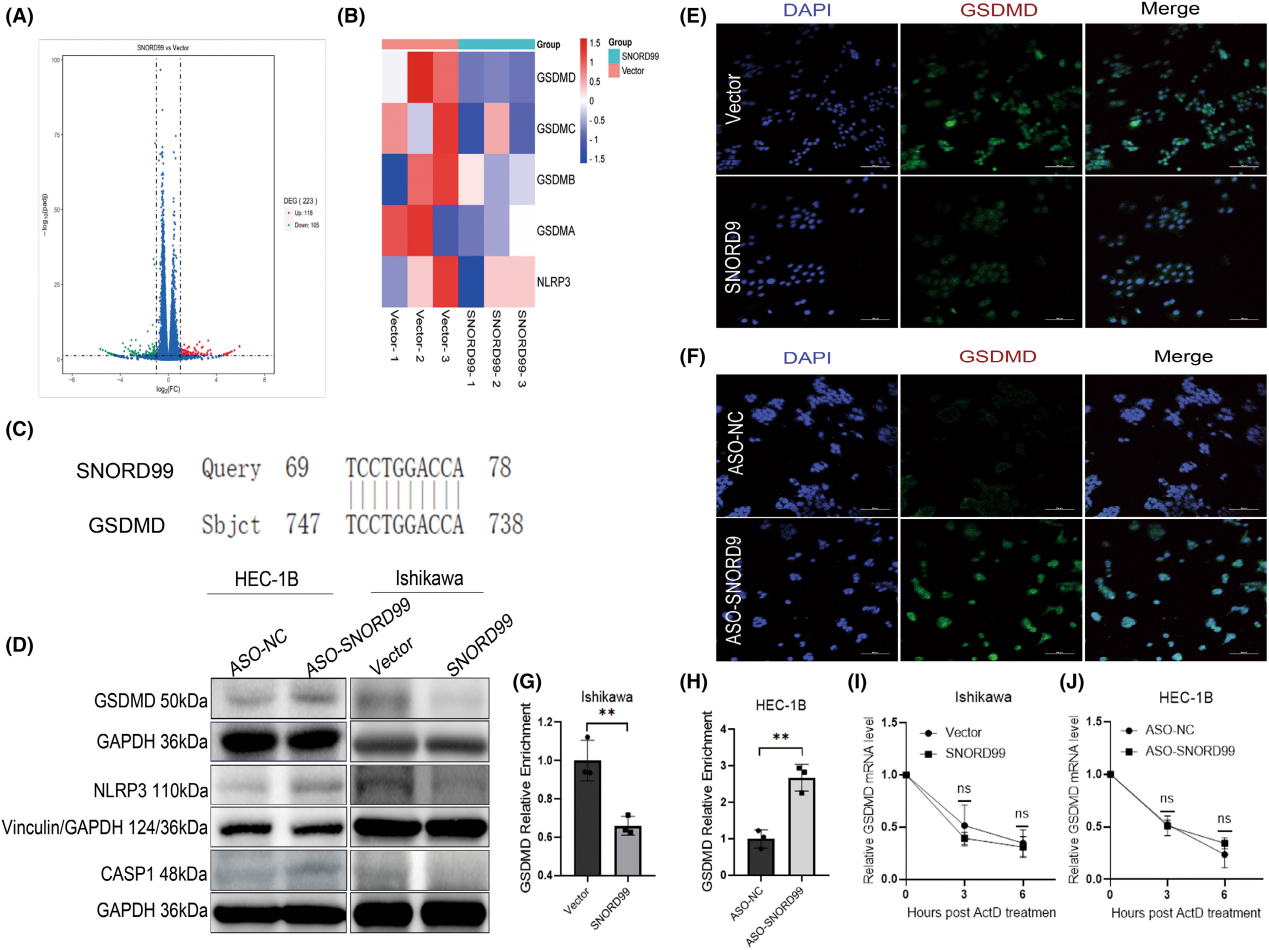
*SNORD99* overexpression inhibits the expression level of pyroptotic proteins in endometrial cancer (EC) cells. (A) Volcano plots of differentially expressed genes exist in RNA‐seq (*n* = 223). (B) Heat map of differentially expressed genes of pyroptosis exists in RNA‐Seq. (C) Base pairing between *SNORD99* and GSDMD. (D) Expression levels of GSDMD, caspase‐1 and NLRP3 proteins in Ishikawa and HEC‐1B cells after overexpression or knockdown of *SNORD99*. (E) After overexpression of *SNORD99*, the fluorescence intensity of GSDMD protein was weakened. (F) After the knockdown of *SNORD99*, the fluorescence intensity of GSDMD protein was enhanced. (G) GSDMD mRNA levels after overexpression of *SNORD99* (*n* = 3). (H) GSDMD mRNA levels after knocking down *SNORD99* (*n* = 3). (I) GSDMD mRNA stability after overexpression of *SNORD99* (*n* = 3). (J) GSDMD mRNA stability after knocking down *SNORD99* (*n* = 3). Values are presented as mean ± SD. *p ≤ 0.05, **p ≤ 0.01 and ***p ≤ 0.001.

The RNA sequencing analysis of *SNORD99* overexpression revealed that differential expression of 223 genes, with 118 genes upregulated and 105 downregulated (Figure 5A,B, *p < 0.05). Through gene complementarity pairing between the bioinformatics prediction (NCBI database) and the sequence base of *SNORD99*, we identified a potential binding site between GSDMD and *SNORD99* (Figure 5C). As GSDMD is a key protein involved in pyroptosis, we performed overexpression or knockdown of *SNORD99* in EC cells to evaluate its effect on pyroptosis. Western blot and immunofluorescence assays were conducted to determine differences in the expression of pyroptosis‐related genes. The results of western blot experiments showed that the overexpression of *SNORD99* decreased the expression of GSDMD, caspase‐1 and NLRP3 (Figure 5D), while immunofluorescence experiments revealed that *SNORD99* overexpression inhibit GSDMD expression (Figure 5E), while ASO transfection to knockdown *SNORD99* promoted GSDMD expression (Figure 5F). The mRNA level of GSDMD is decreased by overexpression of *SNORD99* (Figure 5G, *p < 0.05). On the contrary, knocking down *SNORD99* increased the mRNA expression level of GSDMD (Figure 5H, *p < 0.05). The RNA stability of GSDMD does not exhibit significant correlation with either overexpression or knockdown of *SNORD99* (Figure 5I,J, *p > 0.05).

### 3.6 *SNORD99* regulates GSDMD expression through 2'‐O‐methylation

**FIGURE 6:**
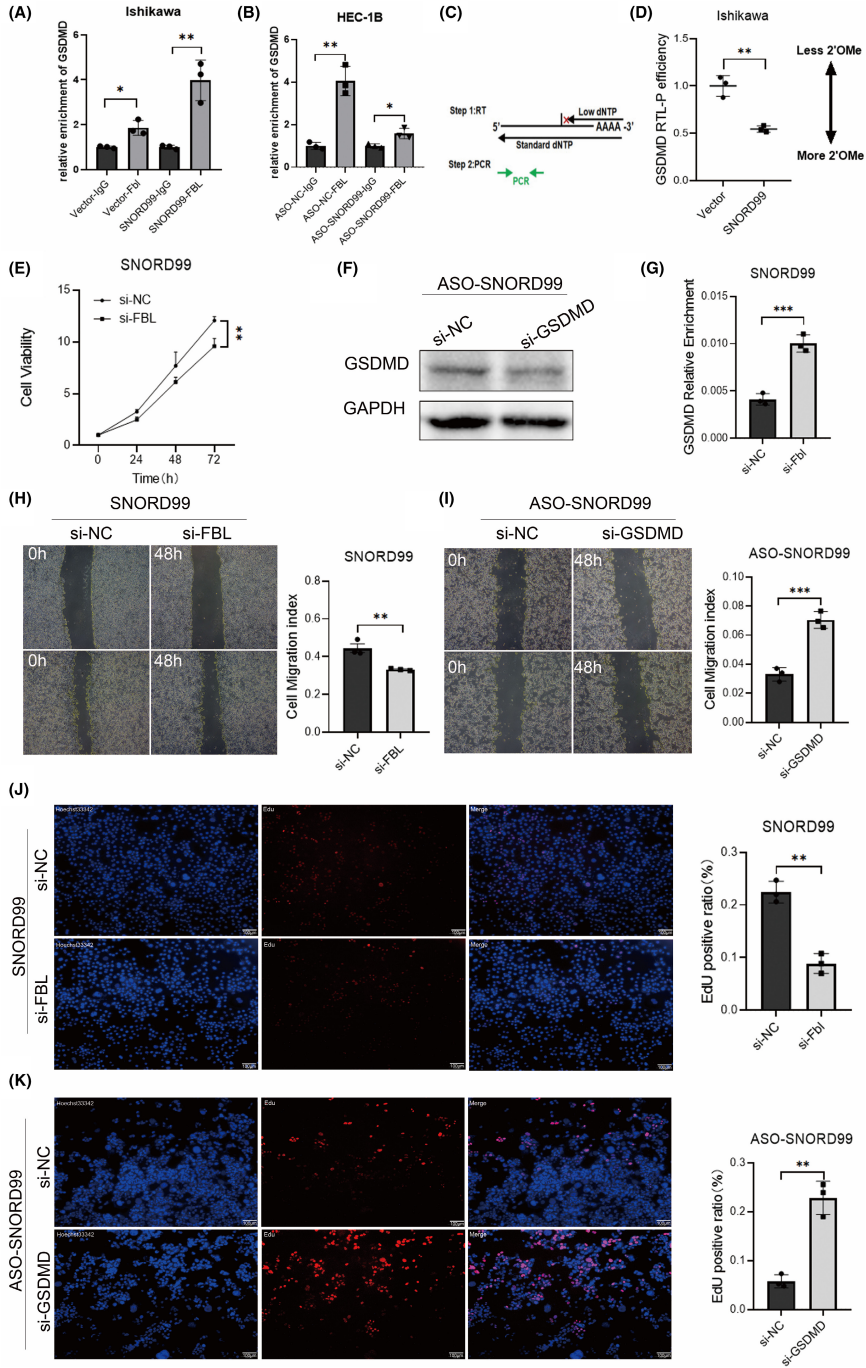
*SNORD99* regulates the 2'‐O‐methylation level of GSDMD. (A, B) FBL can bind GSDMD mRNA and *SNORD99* (*n* = 3). (C) Principle demonstration diagram of RTL‐P experiment. (D) The 2'‐O‐methylation level of GSDMD mRNA in Ishikawa cells overexpressing *SNORD99* (*n* = 3). (E) Cell proliferation of knocking down FBL after over‐expression of *SNORD99* (*n* = 3). (J) EdU fluorescence labelling for cell proliferation of knocking down FBL after over‐expression of *SNORD99* (*n* = 3). (F) Level of GSDMD protein after downregulating the expression level of *SNORD99* before knocking down GSDMD (*n* = 3). (G) mRNA level of GSDMD after overexpression of *SNORD99* and subsequent knockdown of FBL (*n* = 3). (H) Cell migration of knocking down GSDMD after downregulation of *SNORD99* (*n* = 3). (I) Cell migration of knocking down FBL after over‐expression of *SNORD99* (*n* = 3). (K) EdU fluorescence labelling for cell proliferation of knocking down GSDMD after downregulation of *SNORD99* (*n* = 3). Values are presented as the mean ± SD. *p ≤ 0.05, **p ≤ 0.01 and ***p ≤ 0.001.

To establish whether *SNORD99* regulates the expression of GSDMD by affecting the level of 2'‐O‐methylation modification, we first confirmed that both *SNORD99* and GSDMD can bind to 2‐O‐methyltransferase FBL, we conducted RIP experiments after overexpression or knockdown of *SNORD99*. The results indicate that overexpression of *SNORD99* leads to an increase in the binding ability of GSDMD mRNA to the *SNORD99*‐FBL complex, whereas knockdown of *SNORD99* results in a decrease in the binding ability of GSDMD mRNA to the *SNORD99*‐FBL complex (Figure 6A,B, *p < 0.05). Then, we conducted RTL‐P experiments, with specific experimental methods based on existing literature.^20^ The experimental principle of RTL‐P is based on the premise that low dNTP concentrations would terminate cDNA synthesis by reverse transcriptase when encountering 2'‐O‐methylated nucleotides (Figure 6C). The results from RTL‐P revealed that *SNORD99* overexpression led to a significant decrease in the quantity of the GSDMD product under low dNTP concentrations compared to the control group, indicating an increase in the level of 2'‐O‐methylation (Figure 6D, *p < 0.05). These results suggest that *SNORD99* might exert its regulatory effect on GSDMD expression through 2'‐O‐methylation modification. Our results showed that FBL downregulation in *SNORD99* overexpression Ishikawa cells reversed the oncogenic role of *SNORD99*, which was validated by CCK8, wound healing, EdU assays (Figure 6E,H,J, *p < 0.05), and the mRNA expression level of GSDMD is also increased (Figure 6G, *p < 0.05). After further downregulating the expression of GSDMD in HEC‐1B cells with ASO‐mediated knockdown of *SNORD99*, we observed enhanced proliferation and migration capabilities of EC cells (Figure 6I,K, *p < 0.05), accompanied by a corresponding decrease in GSDMD protein expression levels (Figure 6F, *p < 0.05).

### 3.7 *SNORD99* participates in the process of pyroptosis

**FIGURE 7:**
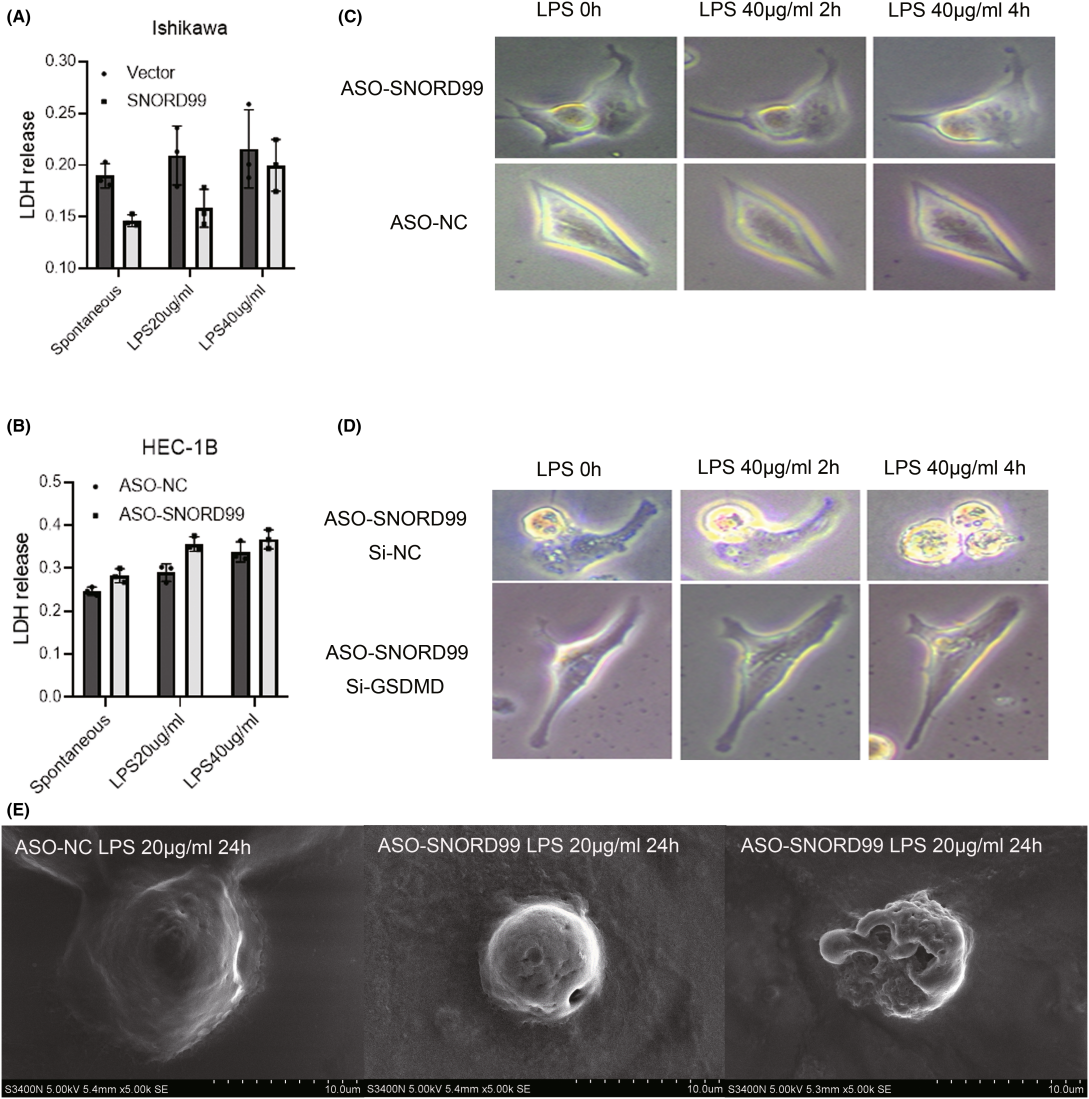
Effects of overexpression or knockdown of *SNORD99* on cell morphology. (A) Lactate dehydrogenase (LDH) levels of Ishikawa cells overexpressing *SNORD99* after treatment with different concentrations of lipopolysaccharide (LPS) (*n* = 3). (B) LDH levels of *SNORD99*‐knockdown HEC‐1B cells treated with different concentrations of LPS (*n* = 3). (C) The morphological changes of HEC‐1B cells knocked down by *SNORD99* after treatment with the same concentration of LPS at different time periods. (D) The HEC‐1B cells that knocked down *SNORD99* and then the expression of GSDMD showed changes in cell morphology at different time periods after treatment with the same concentration of LPS. (E) The cell morphology changes observed by SEM after the HEC‐1B cells knocking down *SNORD99* were treated with the same concentration of LPS. Values are presented as the mean ± SD. *p ≤ 0.05.

LDH is a well‐known marker of cell damage, and it is released from the cell when pyroptosis occurs, along with cytokines, through the cell pores. After the Ishikawa cells overexpressing *SNORD99* were treated with 20 or 40 μg/mL LPS for 24 h, the LDH level in the supernatant of the cell culture medium was significantly lower than that of the control group (Figure 7A, *p < 0.05). In contrast, the knockdown of *SNORD99* expression in HEC‐1B cells led to a significant increase in LDH level in the supernatant compared to the negative control group (Figure 7B, *p < 0.05). Under the light microscope, HEC‐1B cells transfected with ASO *SNORD99* without LPS treatment had a normal appearance, including filamentous protrusions. After treatment with 40 μg/mL LPS for 2 h, the cells became larger and slightly swollen, and the processes were shorter. After 4 h of LPS treatment, the cell processes were much shortened and the cell bodies were significantly swollen (Figure 7C). While knocking down the expression of GSDMD on the HEC‐1B cells that have knocked down the expression of *SNORD99*, it can be observed under the light microscope that the characteristic changes of cell pyroptosis are reversed. In contrast, there was no relevant performance in the control group (Figure 7D). Next, the detailed surface morphology of HEC‐1B cells was evaluated by SEM. After the cells were transfected with ASO to knock down the expression of *SNORD99* and treated with 20 μg/mL LPS for 24 h, many pits or pores of different sizes were observed on the cell surface, the cell shape collapsed and the protrusions shrank (Figure 7E). Conversely, no such manifestations were detected in the negative control group.

## 4 DISCUSSION

In an effort to identify potential biomarkers for EC diagnosis and treatment, we conducted an extensive search for various snoRNAs using the specific database. Our analysis revealed that *SNORD99* expression was considerably higher in EC tissues when compared to normal endometrial tissues, highlighting its potential as a valuable diagnostic and therapeutic marker for this disease.

To better understand the role of *SNORD99* in EC cells, we performed a series of experiments including both in vitro cell studies and subcutaneous tumorigenesis assays in nude mice. By introducing a *SNORD99* overexpression plasmid into Ishikawa cells, we noted a marked rise in cell growth, coupled with a stimulation of cell migration. However, transfection of ASO in HEC‐1B cells to knock down the expression level of *SNORD99* reversed the results of our cell experiments. Furthermore, the subcutaneous tumour formation experiments conducted in nude mice provided additional evidence to support our findings, as we observed a significant increase in tumour growth following *SNORD99* overexpression, validating its role in promoting tumour growth in vivo. These results suggest that *SNORD99* may play a role in promoting the initiation and progression of EC. Despite our researches indicating the potential involvement of *SNORD99* in EC, the precise mechanism of its action still remains unclear and requires further investigation.

2'‐O‐methylation is the partial methylation of the 2'‐OH in nucleotide ribose, which is the main RNA modification mediated by snoRNA and associated with various cancers. An interesting example of the potential involvement of snoRNAs in cancer is the long non‐coding RNA ZFAS1, ZFAS1 has been demonstrated to enhance 2'‐O‐methylation modification mediated by snoRNA through Nop58 recruitment, and has been linked to the progression and prognostication of colorectal cancer.^21^ The 3′‐terminus of Mir‐21‐5p can be modified by adding a methyl group to the 2'‐O position, which has been shown to impact the stability of the methyltransferase HENMT1 and inhibit apoptosis in lung cancer cells.^22^ Recent studies have shown that snoRNAs have the ability to modulate the expression of target genes, affecting both mRNA and protein levels, through modifications such as 2'‐O‐methylation.^23^, ^24^, ^25^ In general, 2'‐O‐methylation can increase mRNA stability. However, our research indicates that the overexpression or knockdown of *SNORD99* does not affect the stability of GSDMD mRNA. Nonetheless, other studies have suggested that 2'‐O‐methylation modification may impact the spatial structure and flexibility of the RNA chain, disrupting the interaction between the ribosome and the codon–anticodon helix. This disruption can lead to errors in ribosomal recognition and proofreading of homologous aminoacyl tRNA, ultimately resulting in decreased targeted gene expression and reduced protein synthesis.^11^ Based on the results, we believe that *SNORD99* can influence the expression of GSDMD mRNA at the transcriptional level. According to the results of western blot and immunofluorescence, GSDMD protein expression decreased following *SNORD99* overexpression, indicating that *SNORD99* can affect GSDMD protein expression at the translational level. RIP results demonstrated that *SNORD99* can form a complex with the 2'‐O‐methyltransferase FBL, which then binds to GSDMD mRNA. RTL‐P results showed that the 2'‐O‐methylation level of GSDMD mRNA increased after *SNORD99* overexpression. These findings indicate that *SNORD99* can modify GSDMD mRNA through 2'‐O‐methylation. Therefore, we hypothesize that *SNORD99* modifies GSDMD through 2'‐O‐methylation, thereby promoting EC occurrence and development. However, due to technical and conditional limitations, we still lack methods to specifically detect the 2'‐O‐methylation sites of GSDMD, further research is needed in the future.

Unlike apoptosis, pyroptosis is a type of inflammation‐dependent programmed cell death that acts as the body's self‐protection mechanism to remove pathogens from the body.^26^, ^27^ Recent studies suggest that pyroptosis may also be closely related to the proliferation and migration of different cancer cells. It can promote the inflammatory cell death of cancer cells and inhibit their proliferation and migration.^28^ Normally, GSDMD is in a state of self‐inhibition. However, when cleaved by pyroptosis‐related caspase enzymes, GSDMD releases the N‐terminal fragment, which forms a hole in the cell membrane, causing the cell to swell and rupture,^15^, ^16^, ^29^, ^30^ which indicates that GSDMD is involved in pyroptosis. Western blot and immunofluorescence experiments showed that the overexpression of *SNORD99* decreased the expression of GSDMD and the pyroptosis‐related protein caspase‐1 and NLRP3, whereas the knockdown of *SNORD99* increased GSDMD expression. Therefore, we propose that *SNORD99* may affect pyroptosis by reducing the expression level of the GSDMD protein. Moreover, changes in cell phenotype were observed under an optical microscope. Finally, we observed changes in cell phenotype by scanning electron microscopy. Under the optical microscope, we found that the negative control HEC‐1B cells still had clear cell protrusions after their treatment with LPS. The cell protrusions were not shortened and disappeared over time, and the cells did not swell significantly. However, after the HEC‐1B cells with knocked‐down *SNORD99* expression were treated with LPS, the cell protrusions were significantly shortened or even disappeared over time, and the cell bodies would also be significantly swollen. Through scanning electron microscopy, we observed that these cells with knocked‐down *SNORD99* expression became shorter and disappeared after treatment with LPS, and there was a large number of pores in the cell membrane. Therefore, *SNORD99* overexpression may inhibit the process of pyroptosis by targeting and regulating GSDMD, representing a new target for the treatment of cancer.

## 5 CONCLUSIONS

**FIGURE 8:**
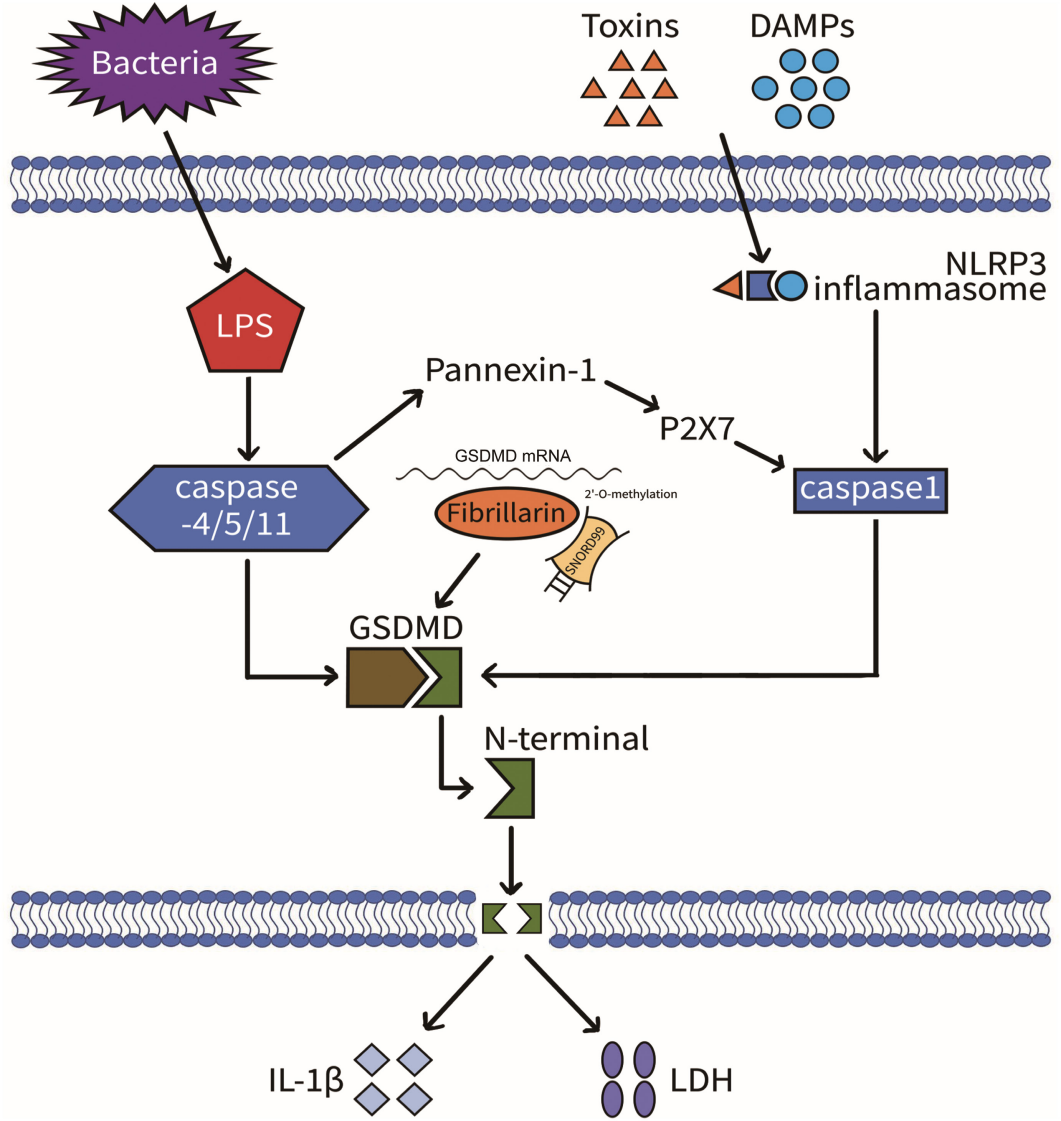
Mechanism diagram of *SNORD99* involvement in cell pyroptosis.

*SNORD99* can downregulate the expression of GSDMD protein by increasing the 2'‐O‐methylation modification level of GSDMD mRNA. Further, it inhibits the process of pyroptosis to facilitate the initiation and progression of EC. Our present findings indicated that snoRNA may be involved in the regulation of GSDMD expression and cell pyroptosis (Figure 8), which provides a new direction in EC prevention and therapy. Therefore, *SNORD99* may become a new therapeutic target for EC.

## AUTHOR CONTRIBUTIONS

**Jing‐yuan Xian:** Conceptualization (lead); data curation (lead); formal analysis (lead); funding acquisition (supporting); investigation (lead); methodology (lead); project administration (lead); resources (equal); software (lead); supervision (lead); validation (lead); visualization (lead); writing – original draft (lead); writing – review and editing (lead). **Wu Wu:** Conceptualization (equal); data curation (supporting); formal analysis (supporting); funding acquisition (supporting); investigation (supporting); methodology (supporting); project administration (supporting); resources (supporting); software (supporting); supervision (supporting); validation (supporting); visualization (supporting); writing – original draft (supporting); writing – review and editing (supporting). **Xi Chen:** Conceptualization (supporting); data curation (supporting); formal analysis (supporting); funding acquisition (supporting); investigation (supporting); methodology (supporting); project administration (supporting); resources (supporting); software (supporting); supervision (supporting); validation (supporting); visualization (supporting); writing – original draft (supporting); writing – review and editing (supporting). **Hai‐juan Bao:** Conceptualization (supporting); data curation (supporting); formal analysis (supporting); funding acquisition (supporting); investigation (supporting); methodology (supporting); project administration (supporting); resources (supporting); software (supporting); supervision (supporting); validation (supporting); visualization (supporting); writing – original draft (supporting); writing – review and editing (supporting). **Song Zhang:** Writing – review and editing (equal). **Xiu‐Jie Sheng:** Writing – review and editing (equal). **Shuo Chen:** Conceptualization (supporting); data curation (supporting); formal analysis (supporting); funding acquisition (lead); investigation (supporting); methodology (supporting); project administration (supporting); resources (lead); software (supporting); supervision (lead); validation (supporting); visualization (supporting); writing – original draft (supporting); writing – review and editing (supporting).

## FUNDING INFORMATION

This work was supported by the National Natural Science Foundation of China (Grant no. 82272985 to Shuo Chen; 82103139 to Song Zhang), and the Project for Key Medicine Discipline Construction of Guangzhou Municipality (no. 2021‐2023‐17).

## CONFLICT OF INTEREST STATEMENT

The authors declare that they have no conflicts of interest.

## Data Availability

The data generated or analysed during this study are included in this published article and its supplementary information files, Supplementary information is available at *Journal of Cellular and Molecular Medicine* website.
